# Solar Photovoltaic and Wind Energy Providing Water

**DOI:** 10.1002/gch2.201600022

**Published:** 2017-07-21

**Authors:** Lawrence E. Jones, Gustaf Olsson

**Affiliations:** ^1^ Monash Energy Materials and Systems Institute (MEMSI) Monash University 20 Research Way, Clayton Campus VIC 3800 Australia; ^2^ Department of Biomedical Engineering Lund University Box 118 SE‐22100 Lund Sweden

**Keywords:** photovoltaic, renewable energy, water‐energy nexus, water supply, wind energy

## Abstract

Renewable energy technologies can make a major contribution to universal access to both energy and water in a sustainable way. In many regions of the world with energy poverty there are abundant renewable energy sources. In this review it is described how solar photovoltaic (PV) and wind energy have a huge potential to supply clean water, in particular in areas with no grid connection. Off‐grid technologies can form a significant part of the solution, all the way from household level to village or community level. Small scale off‐grid systems can provide not only lighting but also energy for pumping to gain access to water and to purify and re‐use water. In rapidly growing peri‐urban areas electric power grids may be available but need to be complemented with decentralized energy sources. Solar and wind can be part of a new kind of hybrid energy supplies. It is noted that there is a confluence of factors, such as greater urbanization, population increase, economic development that will determine the energy mix. The United Nations Sustainable Development Goals of clean water and energy for all are strongly related and will depend to a large extent on solar PV and wind.

## 1 Water and Energy

Renewable energy technologies are already making a major contribution to universal access to carbon free energy. The world now adds more renewable power capacity annually than it adds (net) capacity from all fossil fuels combined.^1^ This will also have a profound impact on access to water. Access to electrical energy can be enjoyed by 84% of the global population implying that almost 1.2 billion people are still without it.^2^ Only in India there are more than 300 million people with no electric power^3^ and the rural electrification rate is only 67%. 91% of the global population has access to an improved drinking water source.^2^ This means that some 650 million people still lack access to clean drinking water.

In many regions of the world with energy poverty there are abundant renewable energy sources. Particularly in Africa and Asia a lot of rural areas are not connected to any grid infrastructure. Solar and wind power offer huge opportunities in off‐grid electricity systems. Small scale off‐grid systems have the potential to improve the energy access in rural and peri‐urban parts of the developing world by providing not only lighting and heating but also pumping to gain access to water as well as water re‐use and purification using different technologies including desalination. Already solar power in small‐scale installations have provided electrical power and lifted millions of people out of poverty.

Electric power grids may be available but need to be complemented with decentralized energy sources in rapidly growing peri‐urban areas. Solar and wind are already becoming part of a new kind of hybrid energy supply. Even in higher‐income countries off‐grid solar is becoming more attractive although in many cases this was due to various policy incentives such as feed‐in‐taxes, and fiscal policy through tax credits.

Available electrical energy is a critical factor to pump or treat water. We will illustrate how solar and wind energy can provide pumping for water supply or irrigation, make treatment of contaminated water sources and water reuse possible. Renewable energy offers new possibilities because of its scalability and can deliver energy for all sizes of water operations, from the household level to the village or urban community level. In particular solar PV is highly modular, from very small lighting systems in remote areas to residential systems and utility‐scale projects; from the kW range to several hundred MWs. Solar PV is also an attractive option for mini‐grids for small villages.^1^

Water and energy for all are among the most essential parts of the 17 UN Sustainable Development Goals (SDG), adopted in 2015 by the international community as part of the 2030 agenda for sustainable development.^4^ The strong couplings between SDG6 (access to affordable, reliable, sustainable and modern energy for all) and SDG7 (clean water and sanitation) are increasingly recognized.^5^, ^6^ The energy‐water‐food nexus is a growing concern for decision makers globally.^3^ Pumping and water treatment by biological processes or desalination will increase the supply of clean water. Conventional electric power technologies, such as thermal power plants, consume large volumes of water for cooling, while solar PV and wind generation have negligible water consumption.

The combination of population increase, climate change and urbanization are strongly related to the availability of energy and water. Having access to energy and clean water is crucial to also to achieve other SDGs:

- SDG1 (No poverty): affordable energy is a prerequisite to eliminate poverty. Today poor people pay a large portion of their income for fuel;
- SDG2 (Zero hunger): pumping for irrigation can be powered by solar and wind;
- SDG3 (Good health and wellbeing): clean water and less air pollution will improve health conditions for the poor;
- SDG5 (Gender equality): today mostly women in rural areas with no access to water are responsible for carrying water. Making water more readily available will give them new opportunities for supporting the family.
- SDG13 (Climate action) is most apparently coupled to renewable energy. Using solar and wind for pumping and desalination can provide water without causing large carbon footprints. In regions with large solar resources, as in the Middle East, desalination with reverse osmosis (RO), relying on solar PV produces water at a price that is lower or equal to fossil fuels.^7^
- SDG14 (Life below water): Solar and wind energy will replace or reduce the consumption of fossil fuels. This will reduce the risks for fuel spills in water bodies.^8^

In the rest of the paper we review solar PV and wind power expansion and their cost development. Renewable energy is also becoming more economically attractive since the costs of conventional systems increase, commitments to reducing greenhouse gas emissions are implemented and targets for exploiting renewable energy are set. The circumstances in rural and remote areas as well as peri‐urban regions are described. Various examples are given how renewable energy is applied for pumping, irrigation, water treatment and re‐use. Land use is an important issue. Comparisons are made to centralized energy and water supplies. In all cases the scalability of both energy generation and water operations is emphasized. Water and energy can be co‐located. Finally future perspectives are sketched.

## 2 Solar PV and Wind Development

Renewable energy operations have several benefits, especially for remote areas: decentralization makes the systems scalable, energy can be provided without a wide‐area power grid, and there is no need to transport fossil fuels for diesel generators. As a result small scale water operations can get their energy requirements satisfied at prices that are becoming affordable even for poor people.

Wind and solar PV made up 90% of the global investments in electric power in 2015^9^ and are now competitive with conventional electric power generation. The cost of wind turbines have fallen by some 40% since 2009 while the price of solar PV has been reduced by 80%. On‐shore wind is now competitive or cheaper than coal, oil and gas‐fired power stations, even without financial support and despite relatively low oil prices.^9^ Still this does not take into account the large social costs of pollution from extracting and burning natural gas or coal.

Solar PV energy systems have been growing at a remarkable rate during the last few years, an exponential growth. At the end of 2015 the global installed solar PV capacity had reached around 227 GWe (gigawatt electric)^2^ (other sources^9^ say 219 GW). Solar PV has achieved cost declines of 40–75% in leading markets since 2010 with both utility‐ and decentralized‐scale installations.^2^ This does not include the costs for balancing the grid system, which will be the next economic challenge for solar and wind systems. This consists of the electrical system costs, costs for permission, and installation. In an off‐grid system the cost for energy storage has to be taken into consideration.

The solar PV additions are led by China, India and the United States.^2^ The cost for solar PV could fall another 60% over the next decade.^10^ Even in regions with large resources of fossil fuel utility scale solar PV projects are competitive with new fossil fuel generation.^10^ There is a dramatic change of the electric power system when the consumers can produce for their own needs. Solar PV is now often the lowest cost option for remote or off‐grid installations.

Solar PV has another advantage. Project lead times are among the shortest of any power generation technology and can be deployed much more rapidly than many other generation options. With the urgent need in many rural and remote areas as well as in many urban areas with frequent blackouts and brownouts the possibility to rapidly install solar PV is a significant benefit.

The global wind power reached 432 GW at the end of 2015^2^ (other sources^9^ say 417 GW), consisting of 420 GW on‐shore and 12 GW offshore. With the current wind power plans the global wind capacity is expected to increase from 432 GW in 2015 to 977 GW in 2030 with about 93% on‐shore.^2^ Wind capacity additions are led by China and the European Union. China's COP21 commitment indicated that wind capacity is to be expanded to 200 GW by 2020 and solar power to 100 GW. There are already signs that China is pushing to higher targets for 2020: a possible 30–50 GW increase for both wind and solar PV.^2^ This will almost surely have global impact. Small‐scale turbines are used for a variety of applications, including rural electrification and water pumping. They are installed increasingly to displace diesel in remote locations.^11^ In the five largest small wind countries the upper capacity limit of small wind is anywhere between 15 and 100 kW.^11^

Both wind power and solar PV use negligible water for electric power production. The major water requirement comes from the manufacturing of the PV cells, the wind mills and the maintenance of the equipment.

For solar thermal plants, however, there is a significant water footprint like for any other thermal power generation.^6^ Some of the water withdrawal is used to raise steam for the steam cycle, but 85–95% of the water requirement is for cooling. The cooling technology determines the water consumption. Moreover, the water quality of the returned water is changed, primarily the temperature. The water demand for once‐through cooling in solar thermal plants is of the same order of magnitude as for coal and nuclear power plants, exceeding some 3.5 m^3^ MWh^−1^. Solar thermal plants with dry cooling has only about 10% water use compared to a system with wet cooling. On the other hand the capital cost is higher and the efficiency is lower,^12^ some 1.5%. Furthermore, a dry cooling system becomes much less efficient at high ambient temperatures.

The public acceptance of solar PV as well as wind is still not trivial, and the attitude of “not in my backyard” is quite common. Another challenge caused by integrating distributed solar PV expansion in existing power grids is the potential impacts on grids operation stemming from the lack of visibility by grid operators into when and the amount of solar power that can be fed onto the system at any given time. On the other hand, experiences around the world show that the cost per kWh of power from large centralized utility‐scaled solar plants is generally much lower than distributed solar. For remote areas outside any power grid it is quite natural that the acceptance is different if it means that the first electric light can be turned on.

### 2.1 Towards Hybrid Energy Generation Mix

Recent advances in small generation technology, increased efficiency and reduction in cost is seeing a push to using more decentralized generation, also in areas with existing power grids. While this trend is set to continue, it is important to note a confluence of other factors. Greater urbanization, population increase, economic development, means that for decades the global energy supply will be in a hybrid state consisting of both centralized and decentralized generation. While the former will be needed for powering large cities, the latter has the potential to serve the energy needs of hundreds of millions of people who will continue to live in rural and peri‐urban areas. As a result, when analyzing the water‐energy nexus, it will be important to develop hybrid policy, regulatory and business models as part of an integrated national energy and water framework. This should also apply to the use of renewable energy.

## 3 Water Supply Using Renewables

Solar PV and wind have an intermittent production. Energy storage technologies, in particular batteries are improving rapidly, since storage is an increasingly important tool for variable renewable energy.^7^ Also in remote areas storage facilities will become essential. In many of these areas, however, peak production times for solar and wind energy aligns with peak demand periods for water pumping and treatment. In many parts of the developing world there is plenty of solar energy while available groundwater may be either brackish (water with salt content of less than 10 000 mg L^−1^) or contaminated. If there is an excess power production this energy can be stored as a clean water buffer or storage for irrigation. In other words, water can serve as important energy storage. The use of solar energy to supply local desalination of brackish water with power is an interesting option where potable water is scarce.

Often solar PV and wind can complement each other to provide a more reliable power source.^13^ Meteorological data will decide whether the cumulated wind and solar energy production can satisfy the load of the plant. However, the combination of solar, wind and battery storage is often too costly for poor areas that may be satisfied, at least primarily, with less ambitious energy supply. The priorities concerning variable energy sources appear quite differently in high‐income and in low‐income regions. In the former case the power availability is emphasized even if it means a higher cost for control and storage. In the latter case there is a higher acceptance that power is not available around the clock.

In regions with existing power grids the links between electric storage, renewable energy and energy efficiency also accentuates a fundamental change that will impact the electric power industry. The rapid growth of distributed generation, particularly rooftop solar, will challenge the conventional utility model. The market changes more rapidly than the regulations.^7^

## 4 Pumping and Irrigation

Solar‐based pumping has already brought cost effective alternatives to grid connected electric pumps or diesel pumps. Naturally pumping service can be brought to areas that are not served by any grid, but also reduces the dependence on grid operation as well as on diesel. In remote or rural areas where energy access is limited or non‐existing and where the water sources are far away then the locals either have to go long distances to fetch the water or to rely on expensive delivery mechanisms such as diesel pumps. In too many peri‐urban areas periodic water deliveries by truck is an expensive water supply.

Among the advantages of solar PV pumping there are four often emphasized: unattended operation, low maintenance cost, easy installation and a long life. Both technology and economic viability have been considered in comprehensive literature reviews of solar pumping technology.^14^, ^15^ The authors have identified factors affecting performance of the solar PV pumping system, the degradation of PV modules as well as efficiency improving techniques. It has been verified that the solar pumping systems are economically viable in comparison to diesel based systems for irrigation and water supplies in rural, remote and in urban regions. The investment payback for some PV water pumping systems has been found to be 4–6 years. The payback time is of course depending on local conditions, as shown below.

The electrical energy required for pumping is dramatically illustrated by the situation in India, where nearly 20% of electricity generation capacity is used for agricultural water pumping.^16^ India has around 26 million agriculture pumps, including at least 12 million grid‐based electric pumps and 10 million diesel operated irrigation pump sets.^17^ Farmers pay only an estimated 13% of the true cost of electricity.^18^ The national burden of electric power subsidies is becoming too heavy. The subsidies encourage inefficient water use and contribute to overdraft of groundwater. As water levels drop, more power is needed to pump the water, thus increasing the energy requirement of water extraction.

India has announced plans to replace many of its 26 million groundwater pumps for irrigation with solar pumps.^19^ This will lead to large savings of installed electric power capacity and of diesel and will reduce huge amounts of CO_2_ emissions. However, it is recognized that solar‐based pumping causes a new risk for water resources. Since the operational cost of solar PV pumps is negligible and the availability of energy is predictable, it could result in overdrawing of water. To combat that unintended consequence, the farmers who accept the subsidies to purchase the solar water pumps must switch to drip irrigation.^19^

In Sub‐Saharan Africa around 40% of the population, more than 300 million people, have no access to an improved source of drinking water from the region.^20^ An analysis of data from 35 countries in sub‐Saharan Africa (representing 84% of the region's population) shows significant differences between the poorest and richest fifths of the population in both rural and urban areas. More than 90% of the richest fifth in urban areas use improved water sources and over 60% have piped water on premises. In rural areas, piped‐in water is non‐existent in the poorest 40% of households, and less than half of the population use any form of improved source of water.

Many parts of the African continent are “energy poor” – electricity service is either intermittent or non‐existent. Some development agencies are looking for solutions by using solar energy to run well systems. Solar PV offers new opportunities as a result of the fast cost reductions. New capacity additions of solar PV in Africa in 2014 exceeded 800 MW, more than doubling the continent's cumulative installed PV capacity. This was followed by additions of 750 MW in 2015.^21^ By 2030 it is expected^7^ that more than 70 GW of solar PV capacity has been installed in Africa. Africa has large solar radiation resources. In the capitals of African countries it ranges between 1750 and 2500 kWh m^−2^ per year while for example in Germany the average irradiation value is just over 1150 kWh m^−2^ per year.^21^

Uganda is implementing a number of projects with the support of development partners and donors that will reduce water costs both in urban and rural areas through use of renewable energy. In the project *Energy for Rural Transformation*, financed partly by the World Bank the 2^nd^ phase is finalizing and the 3^rd^ phase scheduled to be completed in 2020.^22^ The project's objective is to increase access to clean drinking water and also clean affordable energy in schools, health centers and in households. It is recognized that pumping water using energy supply diesel power generators has a high cost. Still Uganda has plenty of sunshine. Uganda is also a beneficiary of a four‐year initiative financed by the Africa Development Bank.^23^ The initiative, ended in 2016, “will contribute to serving an additional 2.4 million people in rural areas and small towns across Uganda,” according to the bank. This includes construction of solar powered water supply systems to replace an estimated 1250 hand‐pumped wells at which people must queue up to get water.

In the Sahel region solar powered pumping stations have been in operation for almost two decades, providing better access to both electricity and water to 2 million people.^24^ The region receives limited annual rainfall and the water table is at most 100 m down. The energy to extract groundwater has helped the people to cope with the prolonged drought conditions. The population in the Sahel regions of West Africa without access to safe drinking water had dropped by 16% during a 10 year period until 2009.

In Kenya only 6% of the agricultural land is irrigated and the main reason for this is lack of energy for pumping. There are some 2.9 million farmers in Kenya. An ongoing project, supported by the Renewable Energy and Energy Efficiency Partnership (REEEP), seeks to implement solar‐powered irrigation.^17^ A typical system can pump up to 20 m^3^ per day and operate at depths less than 15 m. The capital cost for the system is around US$ 400. Considering the savings of fossil fuels the pay‐back time is estimated to two years. The program aims at 30 000 pumping systems by 2018. There are positive social consequences. For example, women and children are relieved from manual pumping and carrying water. As in India, there is an apparent risk of groundwater overdrawing due to the negligible operational cost of PV pumps.

The costs for the systems are significantly different depending on the scale, purpose and configuration of the systems.^7^ Therefore it is more relevant to compare the per‐watt costs of solar PV systems to their direct peers. It may be more meaningful to calculate the cost of the energy services provided and compare that to the existing costs that the user will pay for energy services off‐grid.

In many regions there are long distances between the water source and the consumers. Large‐scale inter‐basin water transfers are common in water scarce regions, where notable examples are the major water transfers between northern and southern California, and between the Yangtse River and northern China. The transfer of large water volumes usually means that large electric energy inputs are needed to supply the pumping systems. This in turn means that the energy requirement for water supply is large. Similarly, to deliver large volumes of water from underground aquifers usually requires significant energy supply. When electricity is delivered via a power grid the issue is about pricing and environmental impact of the energy supply.

## 5 Water Supply by Desalination

Desalination is more and more used as a solution to meet the growing water demand in the world. Over the last 30 years the installed reverse osmosis desalination capacity has increased exponentially. In 2015 there were almost 19 000 desalination plants in operation with a total capacity of 87 million m^3^ d^−1^.^25^ This corresponds to 12 liters of water each day for every one of the 7 billion people on Earth. Desalination requires more energy than any other water supply method,^6^ 3.5–8 kWh m^−3^. Today most of the energy supply for desalination is produced from fossil fuels, and only less than 1% of the energy supply comes from renewables.^26^ Fossil‐fueled desalination has its problems, including the fact that electric power generated from coal and gas plants consume water. Using a water‐intensive resource to produce water is not sustainable. Therefore, we need to think about water as an energy resource and energy as a water resource. The fact that fossil fuel for water production is not sustainable from an economic and environmental point of view has also been recognized in oil rich Saudi Arabia, where the King Abdullah's Initiative for Solar Water Desalination^17^ was announced in 2010. The project has the goal to increase the water security for the country but will also contribute to the development of low cost solar based desalination technology. Increasing scale of deployment will make the solar desalination for affordable in the long term. The cost of input energy is the dominating cost of desalination,^17^ more than 50%.

Besides reverse osmosis there is an emerging technology that is of interest for desalination, membrane distillation (MD).^27^ This is a hybrid membrane‐evaporative process and requires two types of energy, low temperature heat and electricity. Solar collectors and PV panels can be coupled to the MD process. The interest of using solar powered membrane distillation systems for desalination is growing worldwide.^28^ Still, however, the cost of produced water is relatively high compared with that produced from existing solar PV fed reverse osmosis systems.

In order to satisfy water supply in water scarce regions large volumes of water are transported long distances. This is particularly notable when the water is pumped from an inland area to a coastal area. Southern California gets water from the Colorado River and Tripolis at the Mediterranean coast in Libya is supplied by water from the aquifers down south in the Sahara desert. Today desalination using solar PV and wind in the coastal areas instead of exploring inland water and pumping it long distances should be a viable alternative.

### 5.1 Wind‐Powered Desalination

The technical feasibility of wind‐powered desalination with both reverse osmosis and mechanical vapor compression has been studied.^29^ Since 2007 the economics of wind‐powered desalination is even more favorable. However, it should be emphasized that the economics of the wind‐powered desalination process are strongly site‐dependent, so a thorough analysis of local conditions is indispensable.

The city of Perth, Western Australia, started the push for desalination in Australia. The first plant, located in Kwinana, 40 km south of Perth, was put into operation in 2006. The plant produces 150 000 m^3^ d^−1^, which corresponds to some 20% of the city's water supply. An associated wind farm of a 82 MW capacity provides the energy and also produces surplus energy into the grid. A second desalination plant was put into operation in 2011, in Binningup some 150 km south of Perth, with a 50 000 m^3^ d^−1^ capacity in the first stage. Discharging the concentrate from the desalination plant is favorable. There are strong winds and various currents in the Indian Ocean that provide a powerful mixing, which is advantageous for the marine environment when discharging the concentrate from the desalination plant.^30^, ^31^ A desalination plant in Sydney requires 46 MW at full capacity and is powered by 67 wind turbines having a full capacity of 140 MW.^32^ It can supply close to 250 000 m^3^ d^−1^ which is around 15% of all the water needs for the city.

Another example of wind powered desalination is from Texas, USA^33^ in a region suffering from severe water scarcity and depending on deep high salinity aquifers. RO treatment of this kind of brackish water is a realistic and economically feasible solution. There is not only high salinity but arsenic and fluoride concentrations are also high. RO technology would lower these to acceptable limits. A feasibility study has been made for a municipal, integrated wind‐water desalination system for an inland small community. The study from Texas demonstrates that the integration of the two relatively mature technologies of wind energy and RO becomes an attractive match to address an emerging threat to any region heavily dependent on affordable energy and potable water. In the Texas study a small 5‐kW wind turbine provided the energy for a RO desalination plant with the capacity of about 6 m^3^ d^−1^. The energy requirement was found to be around 0.82 kWh m^−3^ of treated water.

The control of the integrated plant uses streaming real‐time water use and electrical demand data in combination with wind speed measurements. Based on the measurements the best use of the energy produced by a turbine array is determined: either for water purification or for displacing conventional power on other municipal loads.

An inland system pumping water from an aquifer differs from a system designed for a coastal location for several reasons. The pumping costs to lift the water from the aquifer to the surface add to the costs of the inland system. Coastal desalination plants are usually located at or near sea level and have minimal lifting costs. The disposal of the brine from the RO process also adds to the cost of the inland system, as coastal locations generally pump the brine back out to sea. However, since the energy need for desalination depends on the salinity there is usually less of an energy cost associated with the purification of brackish aquifer water than sea water.

### 5.2 Solar PV Powered Desalination

Utility‐scale solar is already providing water desalination services and particularly in the Middle East. Environmental impacts, such as greenhouse gas emissions and the by‐products of desalination require careful consideration to balance water security with sustainability.

Solar energy can provide a sustainable alternative to power desalination plants, especially in countries which lie on the solar belt such as Africa, the Middle East, India, and China. Many opportunities appear where both economic and environmental aspects of solar technologies are reviewed.^34^

In the California Central Valley the Panoche Water District is using a solar thermal system for desalination.^35^ Thus, the solar energy is not used to produce electricity. Instead, parabolic trough mirrors turns the solar radiation directly into heat to distill salty water.

In Kotri, a small village of 300 families in the region of Rajasthan in north‐western India a solar‐based RO plant has been put in operation. The plant produces drinking water for more than 1 000 residents from Kotri and surrounding villages.^17^ Brackish water from a nearby lake is pumped through the RO plant and produces around 600 L h^−1^ of water during 6 h every day. The salinity of the water is reduced sufficiently to make the water drinkable. The RO plant is served by a 2.5 kW power plant. The village is in fact connected to the grid, but the supply is very unreliable with only 3 h d^−1^ in most cases. The solar powered system ensures the 6 h electric power supply, which gives some surplus power for light, fans and a computer.

A rural village in India gives a typical example of application of solar PV to treat water.^3^ The SANA organization (Social Awareness Newer Alternatives) identified a village, which had no access to clean drinking water and where the power supply was irregular, the N. Chamavaram village in the state of Andhra Pradesh in south‐east India. Energy from the solar PV system has been used to purify contaminated water to WHO drinking water standard. This is a typical example of decentralized water supply where the raw water intake can be either contaminated well water or used water that is reused. The capacity of this system is 1800 m^3^ of water yearly or 5 m^3^ d^−1^. This will supply 1000 school children from economically backward homes with 5 L of water daily for their families, living in slums nearby.

## 6 Land Use

Land use is a crucial issue in addressing the energy‐water‐food nexus. The competition for land is apparent in several ways. The world is facing an increasing food demand as a result of both increasing average incomes and of the rise in population. At the same time the agriculture yield is feared to drop as a result of water scarcity. It is argued that solar PV and wind power may require fertile land and consequently threaten food production. Actually, solar PV provides a very large benefit by not using land but spare rooftops. The land area requirement for different types of electricity generation can be compared.^6^ Hydropower requires a certain area for the reservoir. Here we assume that the reservoir is used only for hydropower and not for other purposes. For wind power the total area enclosed by the site boundary is considered. Still the area between the towers can often be used for agriculture or forest. Off‐shore wind will of course have an environmental impact as well but the area seldom competes with other uses. Solar PV does not necessarily need to occupy fertile land. Small‐scale PV and solar heating installations have minimal land impact, where they are actively integrated into buildings and structures they serve. The power and energy outputs from a given area are summarized in **Table** **1**. It is obvious that solar PV is very competitive concerning land use, even if the capacity factor is relatively low for the actual area. In the USA the National Renewable Energy Laboratory (NREL) has studied the land footprint of utility scale solar generation. There is a wide range of total land use. The average total land use was estimated to 8.9 acres (around 36 000 m^2^) per MW, or around 28 MW km^−2^ (compare with Table 1).

**Table 1:** Energy output from a given area for different renewable sources^6^

	Hydropower	Wind	Solar PV
Power density MW km^−2^	0.1–17	5–8	20–110
Capacity factor	0.6	0.3	0.2
Annual energy output GWh km^−2^	0.5–90	13–21	35–190

The global share of rooftop PV systems is not known. In many countries there is no separate statistics for rooftop PV and utility scale PV. World Energy Council^3^ reports that only from four major solar PV countries (Germany, Japan, US and Australia) the land savings as a result of rooftop installations exceeds 200 000 acres or 85 000 hectares.

Solar panels are used in innovative ways to save both land and water.^3^ In Japan the 13.7 MW Yamakura floating solar power station is composed of more than 50 000 solar modules, covering a water surface area of 180 000 m^2^. They are mounted on the Yamakura Dam reservoir, located in the Chiba Prefecture, east of Tokyo. The panels will reduce water evaporation from the dam as well as save fertile land. The plant is scheduled to be put in full operation in early 2018.

In India a similar structure is being developed. In a first stage of a solar panel project in the province of Gujarat in north‐western India a 750 m section of a water canal is covered with solar panels, generating 1 MW of electric power. Covering the canal with solar panels will save agricultural land and also decrease the water loss via evaporation.^36^

## 7 Future Decentralization of Water Operations

Just as decentralization has become an economical option for addressing energy access in rural communities, we can expect decentralized production of water to take off in the coming years, not only for rural and remote areas but also for peri‐urban areas. There are a many lessons that the water sector can learn from a decentralized energy sector. Four of these include:

1) The need for new flexible regulatory framework for decentralized water;
2) Tariffs for water should be cost reflective;
3) Business models for water services should factor in potential externalities;
4) Policy and regulation should be designed based on data and evidence, and not politics which could distort the market signals.

Rules and regulations in the electric power area are set up to ensure electricity security and reliable operations. A decentralized water supply has to have a framework that recognizes various scales and local conditions. The regulations also have to consider that the system should reach environmental goals in terms of CO_2_ generation and emissions. The regulations also have to consider the variable power generation.

## 8 Conclusion

For decades ahead there will be development of both centralized and decentralized electric power systems. Here we have emphasized the development of solar PV and wind energy and the consequences for water supply and treatment. We note that:

- Solar PV and wind are getting economically competitive with other energy sources;
- The systems are scalable from the household level and up;
- The systems offer realistic solutions to many regions without any grid connection;
- Decentralized systems may co‐exist with centralized ones;
- The technology gives a potential to satisfy both the water and the energy UN Sustainable Development Goals.

However, it is crucial to remind that off‐grid renewable energy deployment cannot be sustained without technical assistance and human capacity‐building. It requires dedicated measures to identify skills‐related needs and determine how to meet them.

## Conflict of Interest

The authors declare no conflict of interest.
